# Upper airway obstruction following radiofrequency-assisted liposuction of the neck and lower face: a case report

**DOI:** 10.1080/23320885.2022.2128805

**Published:** 2022-09-30

**Authors:** Michèle Chemali, Wassim Raffoul

**Affiliations:** Department of Plastic, Reconstructive and Hand Surgery, Centre Hospitalier Universitaire Vaudois (CHUV), Lausanne, Switzerland

**Keywords:** Airway, liposuction, radiofrequency

## Abstract

Radiofrequency-assisted liposuction (RFAL) recently gained popularity for non-excisional skin tightening. It involves the delivery of a controlled amount of energy resulting in fat liquefaction and skin contraction. It is regarded as safe and effective. We report the case of upper airway obstruction following RFAL of the neck and lower face.

## Introduction

Radiofrequency-assisted liposuction (RFAL) recently gained popularity for non-excisional skin tightening, thereby broadening the plastic surgeons’ armamentarium for specific patients whose skin laxity is not ‘severe’ enough for surgical excision and not ‘mild’ enough for traditional non-invasive esthetic modalities. It is also an option for recurrent skin laxity despite prior surgical excision, and for improving skin laxity in patients who want to avoid surgery and are willing to accept more modest results [1]. RFAL involves the delivery of a controlled amount of energy that is converted to heat, resulting in fat liquefaction, hemostasis and skin contraction by tightening the fibroseptal network, while promoting new collagen and elastin formation and diminishing adipocytes [2]. It is regarded as safe and effective with general anesthesia [3] and local tumescent anesthesia in the awake patient [1]. We report the case of upper airway obstruction following a subcutaneous hematoma with diffuse soft tissue hardening and fat necrosis of the neck and lower face following RFAL.

## Case report

**Figure 1.**
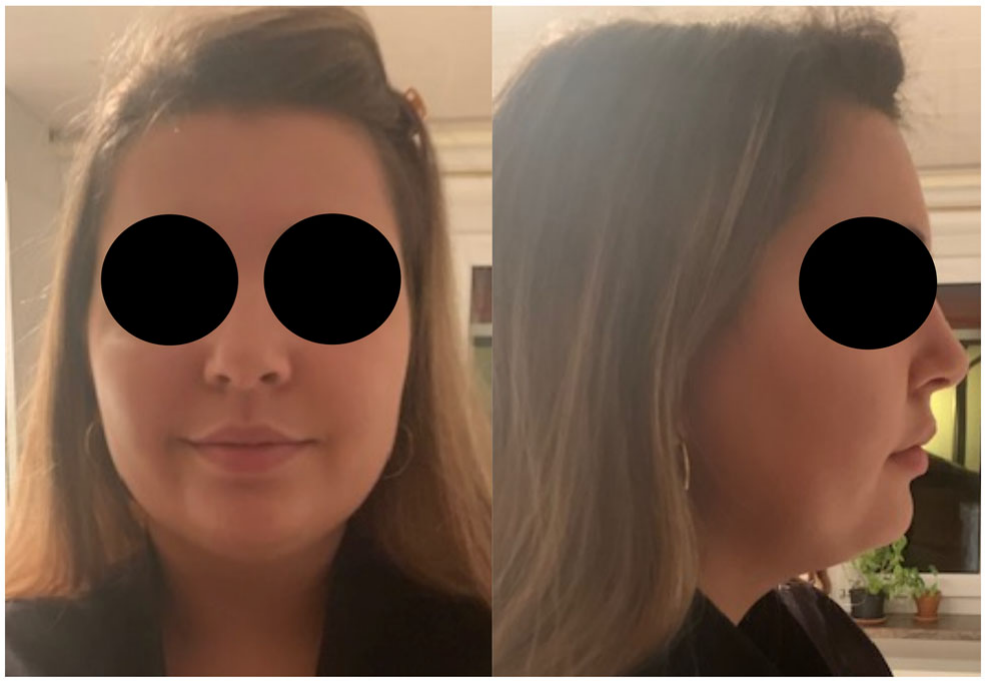
Pre-operative status.

A 21 year-old healthy woman (BMI 25 kg/m^2^) with submental fat excess and poorly defined jawline underwent submental and lower face RFAL technology of Necktite under local anesthesia (Figure 1) in a private clinic by a plastic surgeon well-experienced in RFAL. According to the operative report, local anesthesia with adrenaline was infiltrated in the cervical and submandibular areas (100 cc NaCl 0.9%, 0.2 cc of adrenaline 1 mg/ml, 10 ml of bicarbonate 8.4% and 20 ml lidocaine 20 mg/ml). After liposuction (60 ml), NeckTite was performed. Employing the same radiofrequency energy and liposuction as FaceTitte, Necktite uses a handpiece specific to the neck and jawline. Parameters were set at 0.4 kJ in the anterior neck, 0.2 kJ in the right submandibular area and 0.3 kJ in the left submandibular area (Power setting of 10 W; goal temperature setting for skin heating at 38**°C**). A lightly compressive bandage was then applied.

On postop day (POD) 1, the patient consults the emergency room for severe cervical and lower face edema associated with dyspnea, orthopnea, dysphonia and dysphagia. No signs of local anesthesia toxicity were noted. A CT scan shows diffuse soft tissue edema of the lower face and neck anteriorly and laterally to the level of the sternocleidomastoid muscles and inferiorly to the upper thorax, with no evidence of organized hematoma or associated vascular injury. A nasofibroscopy shows diffuse supra-glottic edema. High-dose intravenous steroids (methylprednisolone 250 mg once, then 200 mg daily for three days, then 125 mg daily), adrenaline aerosols and prophylactic intravenous antibiotics are administered in addition to non-invasive ventilation, and the patient is admitted to the intensive care unit for respiratory surveillance. On POD4, she develops stridor with progressively worsening edema and is therefore intubated; repeat CT scan remains unchanged. On POD6, surgical exploration at the base of the neck reveals diffuse thickening of the subcutaneous tissue, without significant hematoma or abscess formation. Repeat CT scan 24 h later remains unchanged. On POD10, a cervical puncture does not yield any liquid. Immunologic and investigations do not reveal any underlying allergic or immunologic reaction.

**Figure 2.**
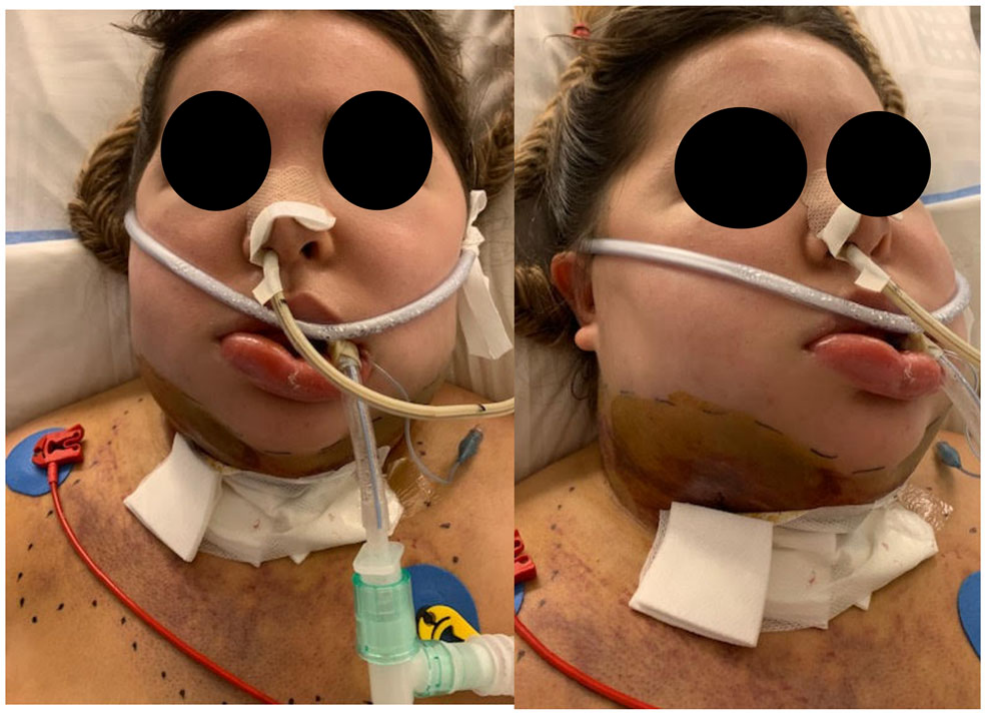
Eleven days postop.

On POD11, the patient is transferred intubated to our hospital university intensive care unit (Figure 2). On physical exam, massive edema and soft tissue hardening were noted at the level of the mid and lower face and neck, associated with skin changes indicating a resorbing hematoma which has migrated from the neck and lower face to the upper chest. Repeat CT scan shows diffuse fat necrosis, with no organized hematoma, vascular injury, collection, abscess formation, thrombosis or mediastinitis. A repeat cervical puncture yields no liquid. Extensive immunologic investigations are repeated and remain negative. On POD15, 1 g of methylprednisolone is administered for one day, followed by 125 mg daily with adrenaline aerosols and topical steroids. She is extubated on POD17.

Nevertheless, on POD21, she develops dysphonia then aphonia with an aggravation of her lower face, neck and supra-glottic edema, necessitating re-intubation. High-dose intravenous methylprednisolone is resumed. Repeat immunologic investigations remain negative. In order to enhance lymphatic drainage, a manual massage was performed twice daily with an intermittent compressive bandage. In addition, a small pillow was placed under the neck to maintain mild extension and avoid neck flexion, which mechanically impedes lymphatic drainage.

**Figure 3.**
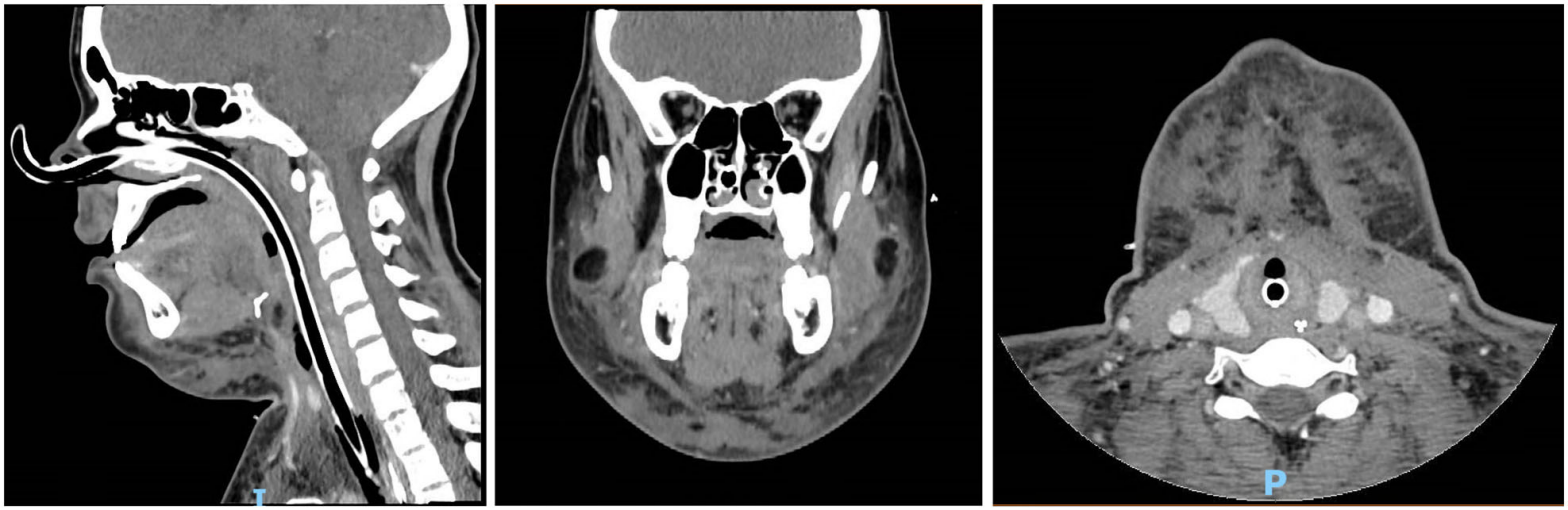
CT scan four weeks postop.

**Figure 4.**
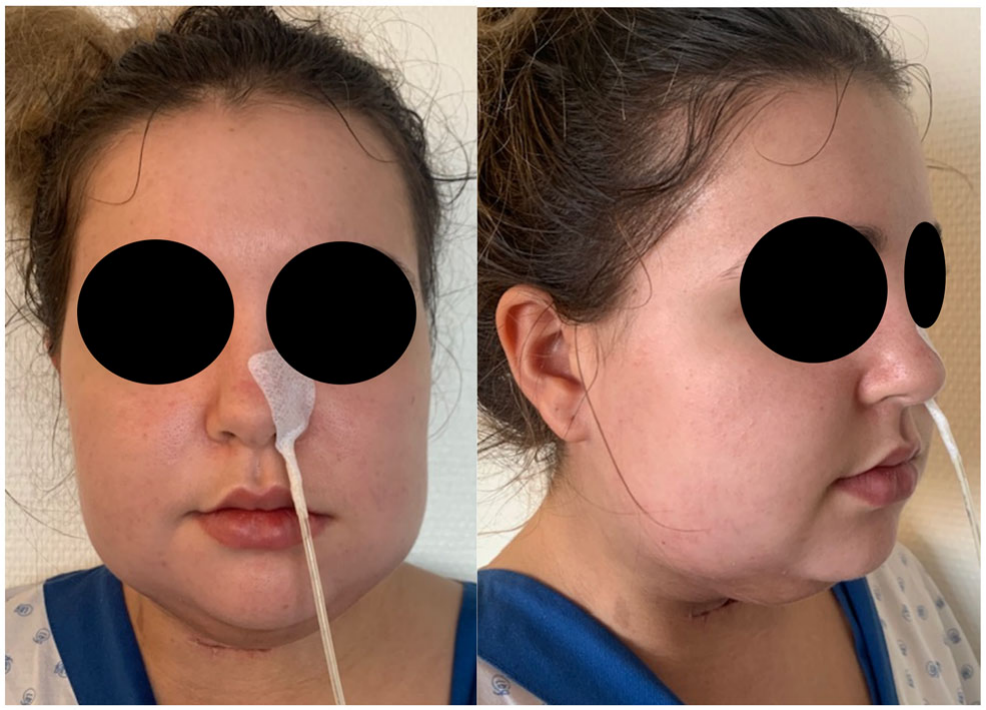
Six weeks postop.

**Figure 5.**
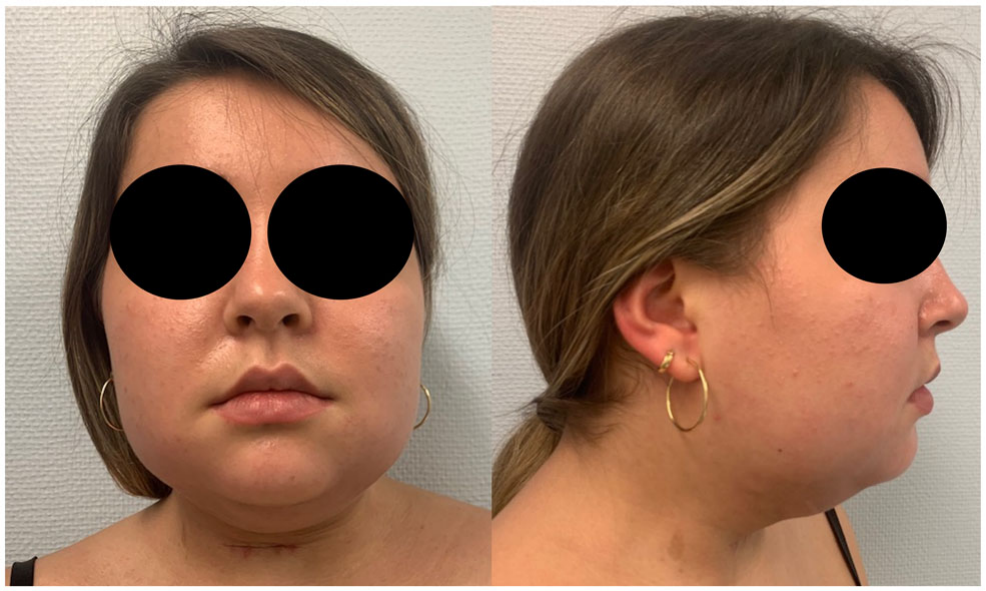
Three months postop.

On POD25, edema begins to subside clinically and on nasofibroscopy &CT scan (Figure 3); the patient is therefore extubated. Oral feeding is started on POD30 and steroids are tapered. She is discharged at six weeks postop (Figure 4). At the follow up visit three months postop, despite daily massage and intermittent compression garments, significant soft tissue hardening and contour deformity persist, associated with bilateral marginal mandibular nerve palsy, submental hypesthesia and dysphonia due to prolonged intubation (Figure 5).

## Discussion

In Pozner et al.’s large series for body and face RFAL of 745 patients under general or local anesthesia, the two most common adverse events were temporary swelling (9%) and nodules (8.5%), which were self-limited. Other minor complications included hyperpigmentation (0.5%) and contour irregularity (1%). No major complications were noted, highlighting the unusual nature of the complication we report. In addition, the authors performed a retrospective chart review whereby nine of the initial 100 patients had retreatment of selected areas, while the final 100 patients sampled did not have any retreatments, thereby suggesting that there may be a learning curve to RFAL [4].

In Theodorou et al.’s series of RFAL of the neck and lower face of 247 patients under general or local anesthesia, complications include prolonged swelling >6 weeks (4.8%), hardened area >12 weeks (3.2%) and marginal mandibular neuropraxia (1.2%), which all resolved without further intervention. There were no repeat treatments or cases requiring operative interventions following RFAL [5].

In Rodopoulo et al.’s series of RFAL of the neck and lower face of 55 patients, 5 (9.1%) patients developed temporary induration and firmness of the soft tissue of the neck that remained for about three months, but fully recovered by persistent, daily massage. They think that the superficial application of high temperature can create this type of hardening, which could become frustrating for the patients and for the doctors to resolve. They recommend smaller energy up to 15 W and deeper application of the device in order to prevent the hardening of the tissue, while still allowing energy to be transferred through the fibroseptal network to the subdermal plexus so that tightening can be achieved [6].

In Chia et al.’s series of RFAL for body contouring in 97 patients with 144 anatomic areas under local anesthesia, the overall complication rate was 14.6% and was not statistically significantly different among the anatomical areas treated. Major complications (6.25%) included infections, seromas, adverse effects from medications, or clinically significant burns outside of the entry sites requiring intervention. Minor complications (8.3%) included periportal burns or end hits that required no intervention. No deaths or hospitalizations were reported in this series. The authors believe that a slower and gradual heating of soft tissues at a lower power setting results in less tissue hardening and fat necrosis, with better tissue response and patient tolerance. The anatomical area requiring the lowest amount of energy was the neck 2.1 kJ. Only one neck case was included in the series, with no complications. Furthermore, the authors believe that RFAL with the BodyTite device does have a learning curve [1].

This case report shows that soft tissue hardening and edema induced by diffuse fat necrosis associated with a subcutaneous hematoma in the neck and lower face following RFAL may be so extensive as to completely obstruct the airway and become life threatening. Although repeat CT scans showed no evidence of organized hematoma, we have noted clinical signs of a resorbing hematoma at the level of the skin overlying the lower face, neck and upper chest. We speculate that mechanical liposuction caused a diffuse subcutaneous hematoma that was exacerbated by RFAL. In addition, the hematoma may have been masked by the radiofrequency-induced edema and fat necrosis, therefore obscuring its visibility on radiologic imaging. We also hypothesize that the extent of soft tissue hardening, edema and fat necrosis may be related to the amount of thermal energy delivered by the RFAL device, despite the parameters reported by the operator to be set within the ‘safe range’, with no other complications encountered during that same period of time (machine dysfunction vs. erroneous setting of the parameters). Based on the above discussion, we speculate that a slower and more gradual heating of soft tissues at a lower power setting with minimal controlled bleeding may prove to be a safer approach, especially in the neck and lower face. In view of this life threatening complication, we suggest that the level of safety of RFAL technology of NeckTite be reassessed.
